# Catalogue of the types of the Scarabaeidae in the National Museum of Natural History of Luxembourg (Coleoptera)

**DOI:** 10.3897/zookeys.814.32059

**Published:** 2019-01-08

**Authors:** Francesco Vitali

**Affiliations:** 1 National Museum of Natural History of Luxembourg, Münster Rd. 24, L-2160 Luxembourg, Luxembourg National Museum of Natural History of Luxembourg Luxembourg Luxembourg

**Keywords:** Coleoptera, Scarabaeidae, holotypes, syntypes, National Museum of Natural History of Luxembourg

## Abstract

The types of Scarabaeidae deposited in the collection of the National Museum of Natural History of Luxembourg are reported for the first time along with some historic and taxonomic remarks: *Entypophanabiapicata* Moser, 1913; *Metabolusthibetanus* Moser, 1914 (currently, *Pseudosymmachia*); *Autosericaannamensis* Moser, 1915 (currently, *Maladera*); *Euphoresiaalboparsa* Moser, 1913; *Hybocamentaferranti* Moser, 1917; *Microsericaflaveola* Moser, 1911; *Triodontalujai* Moser, 1917 (currently, *Triodontella*); *Trochalusferranti* Moser, 1917; *Anomalacondophora* Ohaus, 1913 (currently, *Mimela*); *Amaurinaferranti* Moser, 1911 (currently, *Leucocelis*); *Amaurinavittipennis* Moser, 1909; Cetonia (Eucetonia) kolbei Curti, 1914; *Lomapteradichropusviridipes* Moser, 1908; *Cosmovalgusferranti* Moser, 1912.

## Introduction

This paper represents the second part of the catalogue of the types of Coleoptera deposited in the collection of the National Museum of Natural History of Luxembourg (MNHNL); the first part, concerning the Cerambycoidea, was published in a previous article (Vitali 2010). The purpose of these publications is to record and publicize the scientific value of the material preserved at the MNHNL. Most of the types belong to species collected more than a century ago and they are seldom or never reported in scientific publications.

## Material and methods

Images of two types (Figs 9a, 9b, 13a, 13b) were taken with a Nikon D3300 digital camera equipped with a Tokina AT-X M100 Pro D macro objective and annular light. Those of the remaining types were taken with a CMOS Camera mounted on a Keyence VHX 6000 digital microscope equipped with a VHX-S660E free-angle observation system, a VH-ZST 20–2000× dual-zoom objective, 2D/3D image stitching system and stacking system taking 200 images at 2 million pixels of resolution.

## Results

### The collection of the exotic Scarabaeidae

The collection of the exotic Scarabaeidae with over 3800 specimens represent more than 1100 identified species and subspecies and includes 24 types (4 holotypes and 20 syntypes) belonging to 14 species.

Since 1902, private donations from Luxembourgish and foreign entomologists, besides several acquisitions, have formed the present-day collection. Concerning the early and important donations, 23.7% is represented by the specimens collected by Edouard-Pierre Luja (1875–1953) from Mount Morrumbala (Mozambique), Stanley Falls and Kondué (Democratic Republic of the Congo) and João Monlevade (Brazil) between 1898 and 1924 (Ferrant 1911; Luja 1918, 1951; 1953; Heuertz 1954). This Luxemburgish explorer was a great friend of Victor Ferrant (1856–1942), employee, curator and later director of the MNHNL from 1894 to 1924, and he also worked for Belgian companies. This explains why much of his material, including types, is also preserved in the Royal Institute of Natural Sciences, Brussels (IRSNB) and in the Royal Museum of Central Africa, Tervuren (MRACT).

A second contribution (11.7%) comes from British and German specialists (especially Julius Moser, Friedrich Ohaus, Gilbert John Arrow, Karl-Maria Heller and Karl Jordan), who probably exchanged their material for Luja’s duplicates, which may explain why they described new species based on Luja’s specimens in their collections.

Other contributions come from Ferrant himself (7%), Pierre Hastert (4.9%) and the colonial tutor Paul Sausseau (1873–1912), who sent material from Ambositra, Madagascar (3.3%). The Princess Hilda of Luxembourg (1897–1979) donated material (3.7%) from Ituri, Democratic Republic of the Congo. On the labels, she is mentioned as “Princess Hilda-Schwarzenberg” following her marriage with Adolf, 10^th^ Prince of Schwarzenberg.

Nevertheless, the greater part of this collection (28.9%) comes from the acquisitions of the Museum; the majority of them are constituted by the material bought by Ferrant in 1915 (23.8%) and especially those bought after Ferrant’s retirement in 1924 (34%). Most specimens were thought to have been purchased as identified from the Winkler Catalogue, but the German entomologist Eugen Hintz (before WWI) and Luja (after Ferrant’s retirement) are certainly among the sellers.

Concerning the study of this collection, Ferrant (1911) provided a detailed catalogue of all specialists involved in the determination, according to the studied taxonomic group. Regarding the Scarabaeidae, he quoted Moser (1863–1929) and Ohaus (1864–1946), at that time among the best scarab specialists.

### List of the types

### Subfamily Melolonthinae

#### Tribe Melolonthini

##### Entypophana biapicata

Taxon classificationAnimaliaColeopteraScarabaeidae

Moser, 1913

Figure 1

Entypophana biapicata Moser, 1913a: 295 (type locality: “Neu-Bethel, Usambara”).

###### Syntype.

Usambara / Neu Bethel / VII.1903 // *Entypophana* / *biapicata* Mos. / cotype ♀ [handwritten by Moser] // Donateur 1188d / J. Moser, / Berlin II.1917 // 3197, 1♀.

###### Remarks.

This species was described from an unknown number of specimens of both sexes measuring between 18 and 19 mm collected by Henri Dupré at Neu-Bethel, Usambara, in October 1903. The type locality is today named Mnazi and it is located in the Tanga region (Tanzania) between the Mkomazi National Park and the West Usambaras Lushoto Mountain Reserve. Dupré was a missionary in the Berlin-based protestant Bethel Mission, also known as Berlin III, Evangelische Missionsgesellschaft für Deutsch-Ostafrika (Dupré 1906). Most of his material is therefore conserved in Berlin.

The specimen preserved in the MNHNL does not correspond to the original description in the collecting date (July rather than October) but it was collected prior to the quoted type and belonged to the descriptor. Moreover, it carries Moser’s handwritten label of cotype, suggesting that the author donated this specimen to Ferrant and considered it as a cotype.

**Figure 1.**
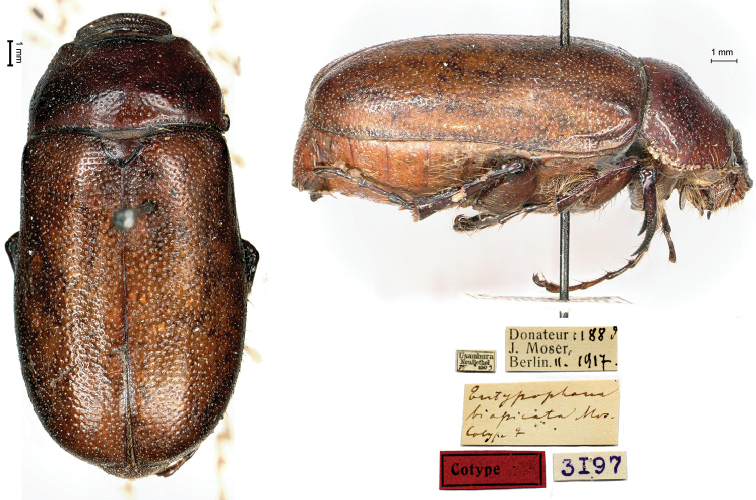
*Entypophanabiapicata* Moser, 1913, syntype. **a** dorsal view **b** lateral view **c** labels.

##### Metabolus thibetanus

Taxon classificationAnimaliaColeopteraScarabaeidae

Moser, 1914

Figure 2

Metabolus thibetanus Moser, 1914: 71 (type locality: “Poo, Thibet”).

###### Syntype.

Thibet / Poo // *Metabolus* / *thibetanus* Mos. / Cotype [handwritten by Moser] // Donateur 944a / J. Moser, / Berlin III.1917 // 2531, 1♂.

###### Remarks.

This species was described from an unknown number of specimens measuring between 10 and 11 mm in body length. The type locality, Poo, also known as Pu, Pooh or Spuwa, is located at 2,662 m altitude in India, Himachal Pradesh, Kinnaur District. The German tibetologist August Hermann Francke (1870–1930) visited this village in July 1910 (Francke 1914: 18) and later, he was appointed professor of Tibetan language at Berlin University. In all likelihood, this is the origin of the entomological material, which Moser described in 1914 and donated to the MNHNL in 1917.

Being preoccupied by *Metabolus* Bonaparte, 1854 (Aves), Dalla Torre (1912) had substituted the genus *Metabolus* Fairmaire, 1887 with *Pseudosymmachia*, but Moser was not aware of this fact.

**Figure 2.**
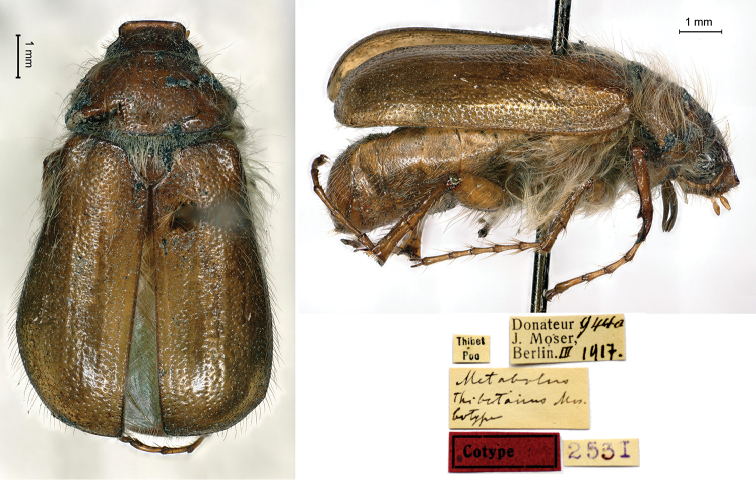
*Metabolusthibetanus* Moser, 1914, syntype. **a** dorsal view **b** lateral view **c** labels.

###### Current status.

*Pseudosymmachiathibetana* (Moser, 1914): Krajcik 2012: 229; Bezděk 2016: 274.

#### Tribe Sericini

##### Autoserica annamensis

Taxon classificationAnimaliaColeopteraScarabaeidae

Moser, 1915

Figure 3

Autoserica annamensis Moser, 1915: 351 (type locality: “Phuc-Son, Annam”).

###### Syntypes.

Annam / Phuc-Son / Nov[ember]-Dez[ember] / H. Fruhstorfer // *Autoserica* / *annamensis* / Mos. [handwritten by Moser] // Donateur 809a / J. Moser, / Berlin III.1917 // 2236, 1♂; ditto, // Donateur 809b / J. Moser, / Berlin III.1917 // 2237 1♀.

###### Remarks.

There are four different localities named “Phúc-Son” in Vietnam, but only one located in Annam belongs to the Anh Sơn District, Nghe An Province. Corresponding to the labels, the German entomologist Hans Fruhstorfer (1866–1922) explored this locality between November and December 1899 (Simon 1903).

**Figure 3.**
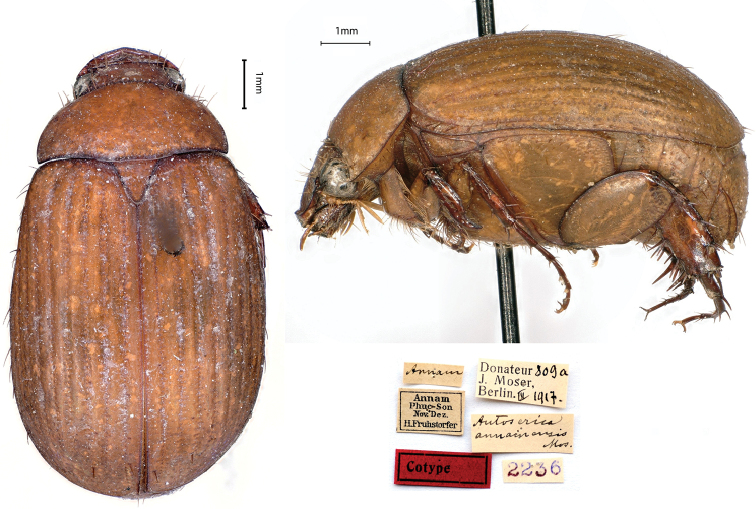
*Autosericaannamensis* Moser, 1915, syntype. **a** dorsal view **b** lateral view **c** labels.

###### Current status.

*Maladeraannamensis* (Moser, 1915): Krajcik 2012: 153.

##### Euphoresia alboparsa

Taxon classificationAnimaliaColeopteraScarabaeidae

Moser, 1913

Figure 4

Euphoresia alboparsa Moser, 1913b: 180–181 (type locality: “Kasai: Kondué”).

###### Syntypes.

Ed. Luja / Kondué / Congo-Belge // Donateur 868a / Ed, Luja, / Lux[em]b[our]g V.1911 // *Euphoresia* / *alboparsa* Mos. [handwritten by Moser] // 2352, 1♂; ditto, Donateur 868b / Ed, Luja, / Lux[em]b[our]g V.1911 // 2353, 1♀.

###### Remarks.

The species was described from an unknown number of specimens measuring 12 mm in body length, which Luja collected during his third mission in Kondué.

The specimens preserved in the MHNL do not show the wording “type” as some of Moser’s other species, but, considering the fact that Moser used different kind of labels, the specimens should be considered as syntypes.

**Figure 4.**
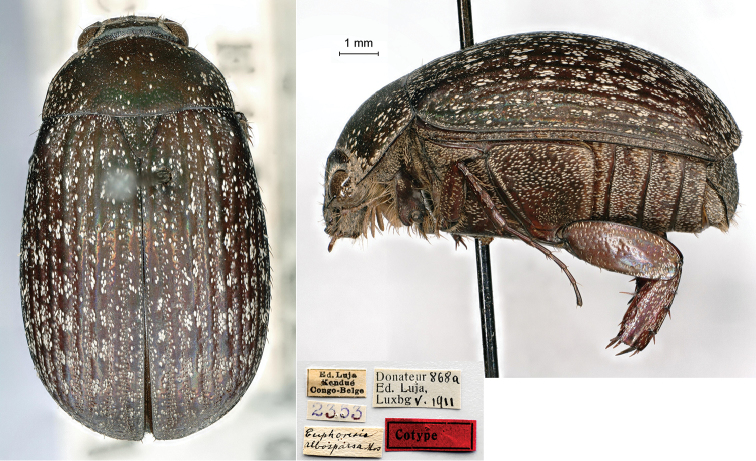
*Euphoresiaalboparsa* Moser, 1913, syntype. **a** dorsal view **b** lateral view **c** labels.

##### Hybocamenta ferranti

Taxon classificationAnimaliaColeopteraScarabaeidae

Moser, 1917

Figure 5

Hybocamenta Ferranti Moser, 1917: 223 (type locality: “Kondué”).
Hybocomenta Ferranti Heuertz, 1954: 32 misspelling

###### Holotype.

Kondué / Kassai / Congo Luja // Donateur 863a / Ed, Luja, / Lux[em]b[our]g V.1911 // *Hybocamenta* / *Ferranti* Mos. Type [handwritten by Moser] // 2342, 1♂.

###### Remarks.

This species was described from a male specimen, measuring 8 mm in body length, collected by Luja during his third mission in Kondué. As for other species, Moser was inaccurate, as he failed to include Kasai in the original description.

**Figure 5.**
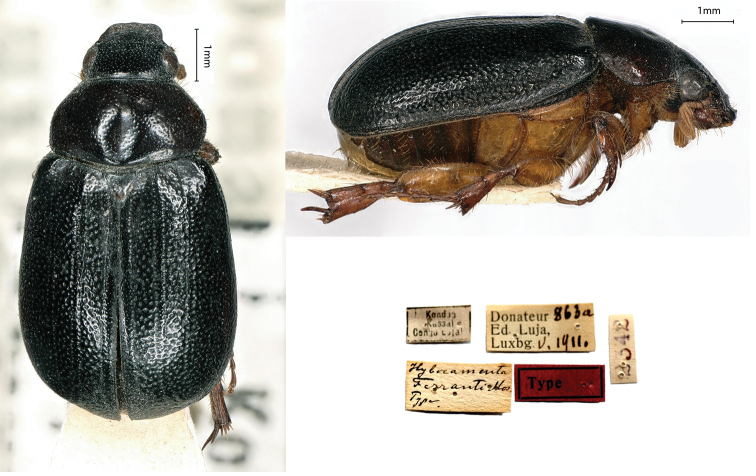
*Hybocamentaferranti* Moser, 1917, holotype. **a** dorsal view **b** lateral view **c** labels.

##### Microserica flaveola

Taxon classificationAnimaliaColeopteraScarabaeidae

Moser, 1911

Figure 6

Microserica flaveola Moser, 1911b: 525 (type locality: “Kina Balu”).

###### Syntypes.

Kinabalu / Borneo, 1500 m / H. Rolle, Berlin, SW. 11 // *Microserica / flaveola* Mos. [handwritten by Moser] // Donateur 826a / J. Moser, / Berlin III.1917 // 2274, 1♂; ditto, Donateur 826b / J. Moser, / Berlin III.1917 //, 2275, 1♀.

###### Remarks.

This species was described from an unknown number of specimens from both sexes measuring 4.5 mm in length. The type locality is Mount Kinabalu, the highest peak in the Bornean Crocker Range (Sabah, Malaysia). The beetles, which Moser had purchased from the well-known German dealer Franz Hermann Rolle (1864–1929), were possibly collected by Fruhstorfer, who explored Mount Kinabalu in 1899.

**Figure 6.**
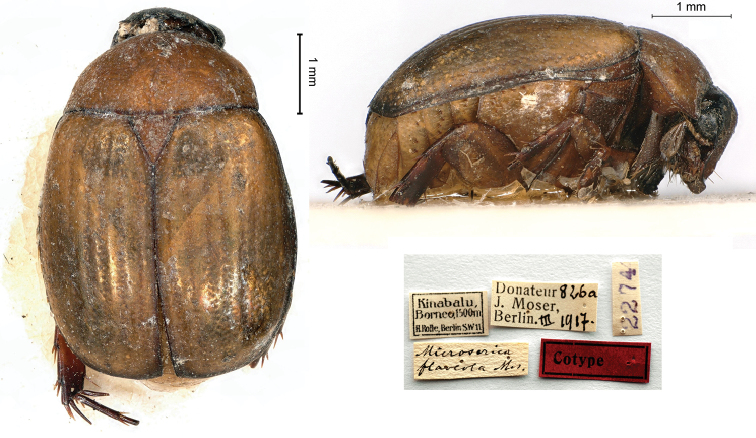
*Microsericaflaveola* Moser, 1911, syntype. **a** dorsal view **b** lateral view **c** labels.

##### Triodonta lujai

Taxon classificationAnimaliaColeopteraScarabaeidae

Moser, 1917

Figure 7

Triodonta Lujai Moser, 1917: 197–198 (type locality: “Kondué, Congo Belge”); Heuertz, 1954: 32.

###### Type.

Ed. Luja / Kondué / Congo-Belge // Donateur 861a / Ed, Luja, / Lux[em]b[our]g V.1911 // *Triodonta* / *Lujai* Mos. / n. sp. [handwritten by Moser] // 2338, 1♂.

###### Remarks.

The species was described from an unknown number of specimens of both sexes measuring from 5.5 to 7 mm in body length. Luja had collected these during his third mission in Kondué.

The genus *Triodonta* Mulsant, 1842, being preoccupied by *Triodonta* Bory de Saint Vincent, 1827 (Colpodidae, Regnum Chromista), was substituted by *Triodontella* by Reitter (1919).

**Figure 7.**
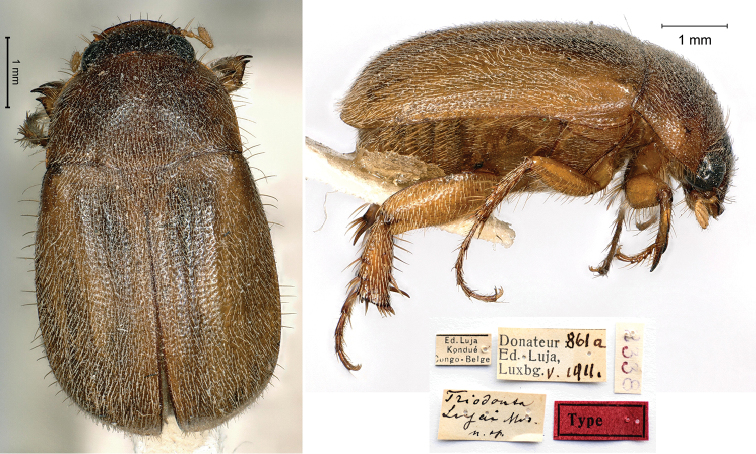
*Triodontalujai* Moser, 1917, holotype. **a** dorsal view **b** lateral view **c** labels.

###### Current status.

*Triodontellalujai* (Moser, 1917): Krajcik 2012: 257.

##### Trochalus ferranti

Taxon classificationAnimaliaColeopteraScarabaeidae

Moser, 1917

Figure 8

Trochalus Ferranti Moser, 1917: 199–200 (type locality: “Kassai, Congo Belge”); Heuertz, 1954: 32.

###### Syntypes.

Ed. Luja / Kondué / Congo-Belge // Donateur 858a / Ed, Luja, / Lux[em]b[our]g V.1911 // *Trochalus* / *Ferranti* Mos. n. sp. [handwritten by Moser] // 2332, 1♂; ditto, Donateur 858b / Ed, Luja, / Lux[em]b[our]g V.1911 // 2333, 1♀; ditto, Donateur 858c / Ed, Luja, / Lux[em]b[our]g V.1911 // 2334, 1♀; ditto, Donateur 858d / Ed, Luja, / Lux[em]b[our]g V.1911 // 2335, 1♀.

###### Remarks.

The species was described from an unknown number of specimens (“ich widme diese Art dem Konservator V. Ferrant in Luxemburg, welcher mir einige Exemplare gütigst überlief”) from both sexes measuring 6 mm in body length. Luja had collected these during his third mission in Kondué.

The type locality is “Kassai” and none of the specimens preserved in the collection of the MNHNL come from this locality. However, Kondué is located in the Kasai region and some specimens in the museum show old labels referring “Kondué / Kassai / Congo, Luja” (see e.g. the following species). Considering Moser’s imprecision in mentioning type data (see *Lomapteraviridipes*), it is likely that “Kassai” included both types of label.

**Figure 8.**
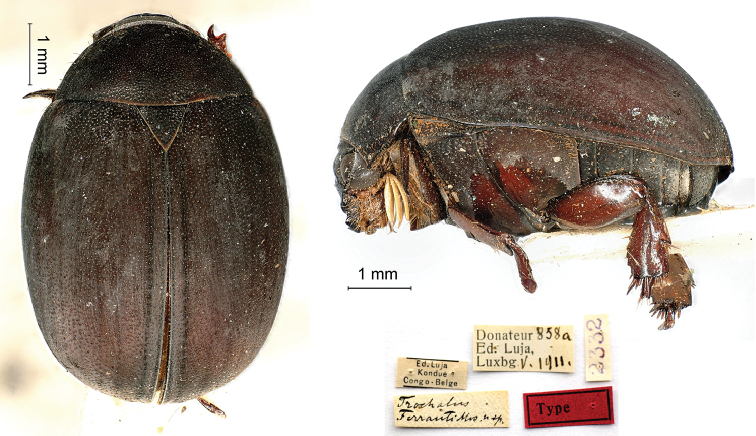
*Trochalusferranti* Moser, 1917, syntype. **a** dorsal view **b** lateral view **c** labels.

### Subfamily Rutelinae

#### Tribe Anomalini

##### Anomala condophora

Taxon classificationAnimaliaColeopteraScarabaeidae

Ohaus, 1913

Figure 9

Anomala condophora Ohaus, 1913: 207 (type locality: “Kondué”).

###### Syntype.

Kondué / Kassai / Congo Luja // Det. F. Ohaus 1912 / *Anomala* ♀. / *condophora* / Ohaus Cotype [handwritten by Ohaus] // Donateur 1025a / Ed, Luja, / Lux[em]b[our]g V. 1907 // 2746 // 1♀.

###### Remarks.

Ohaus (1913) described this species from an unknown number of females coming from Kondué. In the introduction of his article, Ohaus thanked Ferrant and other directors of European museums for allowing him to retain material for his own collection. The handwritten label implies that part of this material was returned to the museums of origin. The date of the label shows that Luja collected part or all these types during his second mission in Kondué. However, besides this cotype, the MNHNL owns 20 other specimens of both sexes, some donated to MNHNL in 1917 after his fourth mission in Kondué. Some of the earlier specimens might also be types.

**Figure 9.**
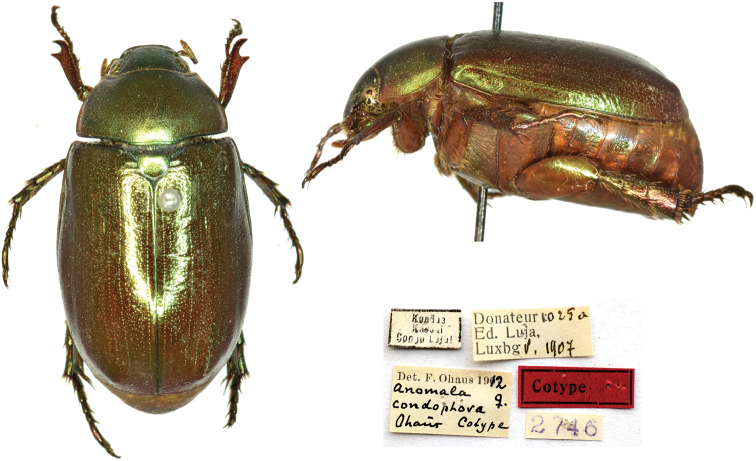
*Anomalacondophora* Ohaus, 1913, syntype. **a** dorsal view **b** lateral view **c** labels.

###### Current status.

*Mimelacondophora* (Ohaus, 1913): Krajcik 2012: 162.

### Subfamily Cetoniinae

#### Tribe Cetoniini

##### Amaurina ferranti

Taxon classificationAnimaliaColeopteraScarabaeidae

Moser, 1911

Figure 10

Amaurina Ferranti Moser, 1911a: 125 (type locality: “Zambèze”).
Amarina Ferranti Ferrant, 1911: 255; Heuertz, 1954: 32 misspelling.

###### Holotype.

M[ont]. Morrumbala / Zambèze 1899 / Ed. Luja // Donateur 1415a / Ed, Luja, / Lux[em]b[our]g VI.1902 // *Amaurina* / *Ferranti* Mos. Type [handwritten by Moser] / J. Moser determ. 1911 [printed] // 3882, 1♂.

###### Remarks.

The collection label contains an incorrect date, as Luja was employed by the Portuguese Company of Zambezi in a coffee plantation on Mount Morrumbala (Mozambique) from spring 1900 to January 1902 (Luja 1951). Heuertz (1954) provided some erroneous data as well by claiming that Luja started his mission in Zambezi in 1901. According to the description (Moser 1911a), another specimen, donated by Ferrant himself, was preserved in the author’s collection.

**Figure 10.**
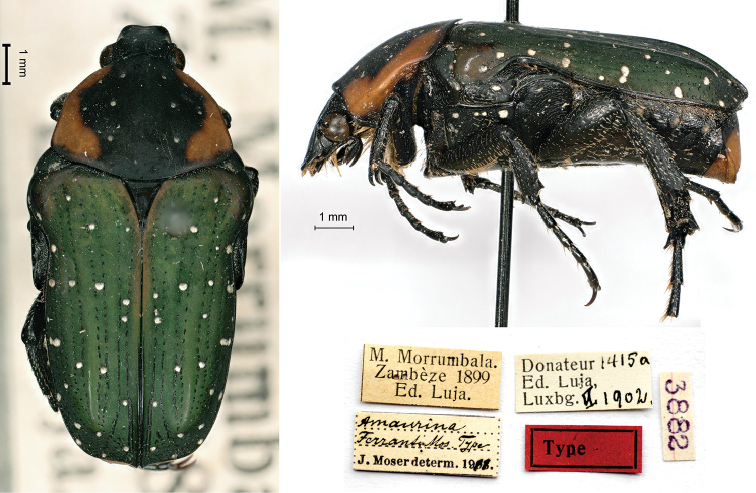
*Amaurinaferranti* Moser, 1911, holotype. **a** dorsal view **b** lateral view **c** labels.

###### Current status.

Leucocelis (Amauroleucocelis) ferranti (Moser, 1911): Krajcik 2012: 142.

##### Amaurina vittipennis

Taxon classificationAnimaliaColeopteraScarabaeidae

Moser, 1909

Figure 11

Amaurina vittipennis Moser, 1909: 323 (type locality: “Sankuru, Kassai”).

###### Syntype.

Sankuru / Congo-Belge 1901 / Ed. Luja // Donateur 1416a / Ed, Luja, / Lux[em]b[our]g V.1911 // *Amaurina* / *vittipennis* / Moser [handwritten by Moser] // 3883, 1♂.

###### Remarks.

The species was described from an unknown number of specimens, measuring 9 mm in body length, which Luja collected in “Sankuru” and “Kassai”. Such localities must be referred to the current provinces Sankuro and Kasai in the Democratic Republic of the Congo, at that time united in the former province Kasai-Oriental.

In spite of the original labels, Luja collected these specimens from August 1898 to 1899, when Baron van Eetveld, general secretary of the Independent State of the Congo, employed him to collect living plants for the Universal Exposition of Paris 1900 (Luja 1951). He provided a long report of this mission that amazed his contemporaries at home (Feltgen 1901), due to major discoveries, especially concerning new species and forms of plants (Ferrant 1911; Heuertz 1954). In 1901, Luja settled in Mozambique (Luja 1951). The label mentioning the donation is wrong as well, as the date is subsequent to Moser’s description.

As for *Euphoresiaalboparsa*, the specimen preserved in the MHNL does not show the wording “type” as some of Moser’s other types, but it should be deemed to be a syntype.

**Figure 11.**
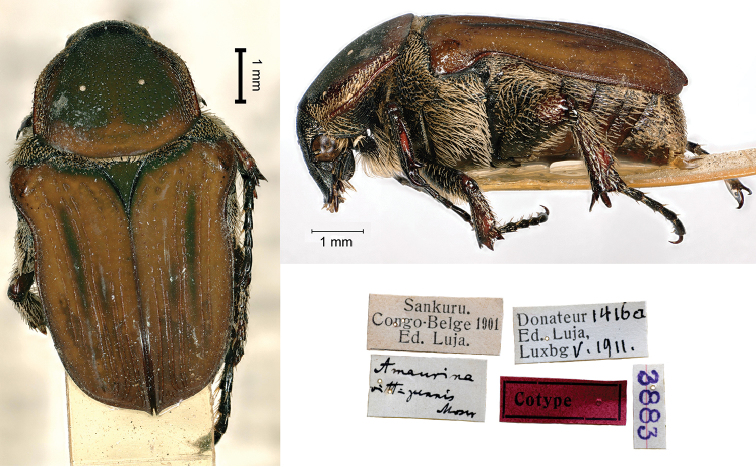
*Amaurinavittipennis* Moser, 1909, syntype. **a** dorsal view **b** lateral view **c** labels.

##### Cetonia (Eucetonia) kolbei

Taxon classificationAnimaliaColeopteraScarabaeidae

Curti, 1914

Figure 12

Cetonia (Eucetonia) kolbei Curti, 1914: 125 (type locality: “Cina, Tsingtau, Linkun”).

###### Syntypes.

Tsingtau / Prof. Hoffmann // Juni // det. Curti / Kolbei m[ihi] // *EucetoniaKolbei Curti* [handwritten by Kolbe?] // Acquisition du Musée / 2352a 1920 // 14220, 1ex.; ditto, Acquisition du Musée / 2352b 1920 // 14221, 1 ex.

###### Remarks.

Both specimens did not have the labels of type but the labels “det. Curti / Kolbei m[ihi]” clearly indicate type material. Curti (1914) described this species from an uncertain number of specimens coming from Tsingtau and originally preserved in the Königliches Museum (now Altes Museum) Berlin. The type locality, currently Tsingtao or Quingdao (Shandong), was a German colony from 1897 to 1919.

Even if not reported in the original description, the types were collected by a certain “Prof. Hoffmann”, which should not be confused with the best-known professor William E. Hoffmann of Lingnan University, Canton (1896–1989). Ten persons were named Hoffmann in the list of the German military present in Tsingtao in that period (Schmidt 2018), but Heinrich Fritz August Wilhelm Hoffmann (1875–1950) is the only professor among them. A medical officer in Tsingtao since February 1913, he was interned in China after the Japanese occupation in November 1914 and released in June 1915. The description occurred in the meeting of the Section Coleopterology of 18 February 1914; thus, the types were certainly collected in June 1913 and sent to Hermann J. Kolbe, curator of the Museum, before the onset of WWI.

*Eucetonia*, originally described as genus (Schoch 1894), was considered a synonym of *Cetonia* (Arrow 1910) or again as a genus (Schürhoff 1942). However, the combination “*Eucetoniakolbei*” was used only by Lucas (1918).

**Figure 12.**
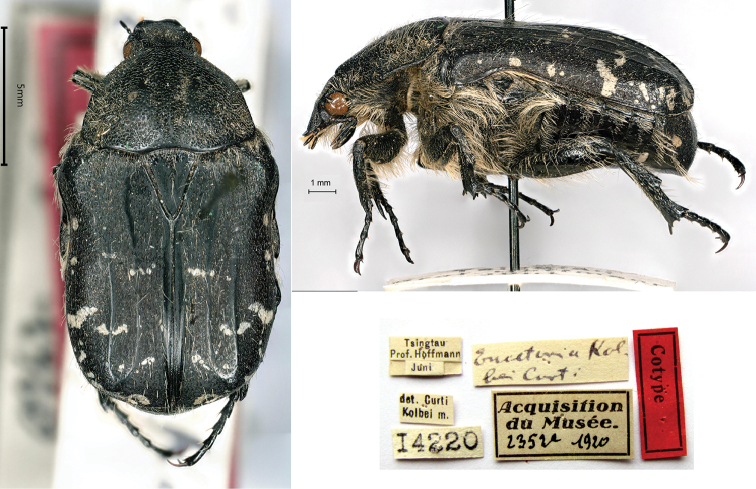
Cetonia (Eucetonia) kolbei Curti, 1914, syntype. **a** dorsal view **b** lateral view **c** labels.

#### Tribe Schizorhinini

##### Lomaptera dichropus viridipes

Taxon classificationAnimaliaColeopteraScarabaeidae

Moser, 1908

Figure 13

Lomaptera dichropus viridipes Moser, 1908: 88 (type locality: “Deutsche Neu-Guinea, Huon-Golf”).

###### Syntypes.

D[eutsche] Neu-Guinea / Sialum [handwritten] // *Lomaptera* / *dichropus* Lsbg. / *subsp. viridipes* Moser / Cotype [handwritten by Moser] // Donateur 1306a / J. Moser, / Berlin III.1917 // 3593, 1♂; ditto, Donateur 1306b / J. Moser, / Berlin III.1917 // 3594, 1♀; ditto, Donateur 1306c / J. Moser, / Berlin III.1917 // 3595, 1♀; ditto, Donateur 1306d / J. Moser, / Berlin III.1917 // 3596, 1♀.

###### Remarks.

The species was described from an unknown number of specimens measuring from 21 to 25 mm. The type locality “Huon-Golf” (currently, Huon Gulf) is the large gulf in eastern Papua New Guinea belonging to Marobe Province. However, Sialum (at one time, Helena-Hafen) is located at north of the Huon peninsula, beyond that gulf.

**Figure 13.**
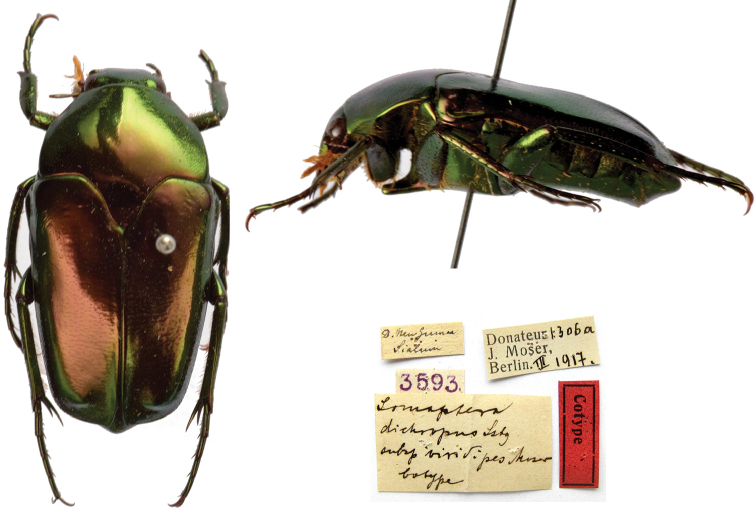
*Lomapteradichropusviridipes* Moser, 1908, syntype. **a** dorsal view **b** lateral view **c** labels.

###### Current status.

Rigout and Allard (1997: 88) treated *L.d.viridipes* as a variety of *Lomapterahelleriana* Valck Lucassen, 1961 without explanation. However, De Jong (1970: 266) and Carter et al. (2016: 6) treated it as *Lomapteraviridipes* Moser, 1908.

#### Tribe Valgini

##### Cosmovalgus ferranti

Taxon classificationAnimaliaColeopteraScarabaeidae

Moser, 1912

Figure 14

Cosmovalagus Ferranti Ferrant, 1911: 255 nomen nudum, misspelling.
Cosmovalgus Ferranti Moser, 1912: 574 (type locality: “Kondué, Congo-belge”); Heuertz 1954: 32.

###### Holotype.

Ed. Luja / Kondué / Congo-Belge // Donateur 1452a / Ed, Luja, / Lux[em]b[our]g V.1907 // *Cosmovalgus* / *Ferranti* Mos. [handwritten by Moser] // 3978, 1♂.

###### Remarks.

Though two males of the collection carry the label of type, the species was described from only one male (“Der vorliegende ♂ ist…”) measuring 10 mm in body length. Luja collected these specimens during his second mission in Kondué. As for *Euphoresiaalboparsa* and *Amaurinavittipennis*, both specimens preserved in the MNHNL do not show the wording “type”, but considering that Moser did not use standardised labels, the one bearing Moser’s label should be deemed as the holotype.

**Figure 14.**
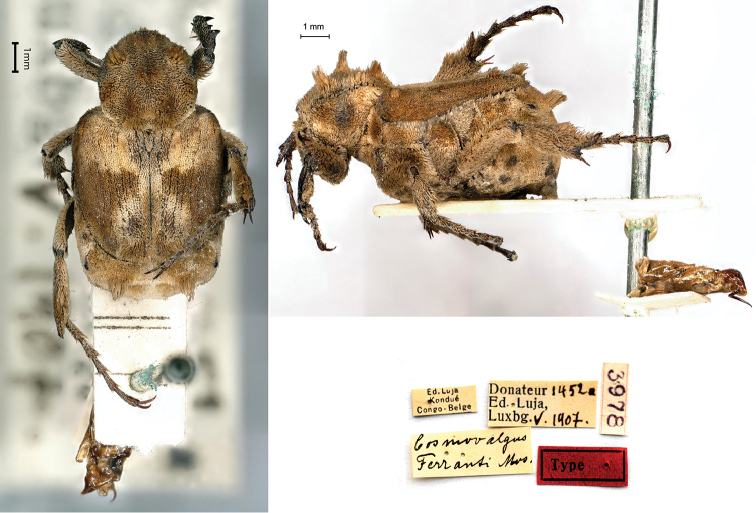
*Cosmovalgusferranti* Moser, 1912, holotype. **a** dorsal view **b** lateral view **c** labels.

### Non-types, even if quoted as such by Heuertz (1954)

#### Bilga pictipennis

Taxon classificationAnimaliaColeopteraScarabaeidae

Fairmaire, 1893

Bilga pictipennis Fairmaire, 1893: 137–138 (type locality: “Oubanghi”); Heuertz 1954: 32.

##### Materials.

Ed. Luja / Kondué / Congo-Belge // Donateur 846a / Ed, Luja, / Lux[em]b[our]g V.1907 // 2311, 1♂; ditto, Donateur 846b / Ed, Luja, / Lux[em]b[our]g V.1907 // 2312, 1♀.

##### Remarks.

These specimens cannot be Fairmaire’s types due to obvious chronological reasons.

#### Autoserica flaviventris

Taxon classificationAnimaliaColeopteraScarabaeidae

Moser, 1916

Autoserica flaviventris Moser, 1916: 246–247 (type locality: “Kassai”); Heuertz 1954: 32.

##### Materials.

Kassai / Kondué / Congo Ed. Luja // Donateur 807a / Ed, Luja, / Lux[em]b[our]g V.1907 // *Autoserica* / *flaviventris* Mos. [handwritten by Moser] // 2229, 1♂; Ed. Luja / Kondué / Congo-Belge // Donateur 807b / Ed, Luja, / Lux[em]b[our]g V.1907 // 2230, 1♀.

##### Current status.

*Maladeraflaviventris* (Moser, 1916): Krajcik 2012: 154.

#### Autoserica ruficeps

Taxon classificationAnimaliaColeopteraScarabaeidae

Moser, 1916

Autoserica ruficeps Moser, 1916: 243–244 (type locality: “Kisantu and Kondué”); Heuertz 1954: 32.

##### Materials.

Ed. Luja / Kondué / Congo- Donateur 806a-e / Ed, Luja, / Lux[em]b[our]g V.1907 // Belge // *Autoserica* / *ruficeps* Mos. [handwritten by Moser] // 2225–2228, 2♂, 2♀.

##### Current status.

*Maladeraruficeps* (Moser, 1916): Krajcik 2012: 155.

#### Euphoresia kassaiensis

Taxon classificationAnimaliaColeopteraScarabaeidae

Moser, 1916

Euphoresia kassaiensis Moser, 1916: (type locality: “Kondué, Kassai”); Heuertz 1954: 32.

##### Material.

Ed. Luja / Kondué / Congo-Belge // Donateur 867a / Ed, Luja, / Lux[em]b[our]g V.1911 // *Euphoresia* / *kassaiensis* Mos. [handwritten by Moser] // 2351, 1♂.

##### Remarks.

In the last three cases, the true types were sent for identification by Henri Schouteden, who worked for the Royal Museum for Central Africa, Tervuren, Belgium (Moser 1916: 233). Hence, all specimens preserved in the MNHNL are simply topotypes.

## Remarks

Analysing the presence of the type material in the collections of the exotic Coleoptera preserved in the MNHNL, the collection of Scarabaeidae shows a far fewer number of types in comparison to that of Cerambycoidea (Vitali 2010). Despite the larger number of exotic scarabs (3825 vs 3221 cerambycids), the number of types is remarkably fewer (24 vs 163). This is not due to the minor productivity of the concerned specialists; Ohaus is considered the “Father of Rutelinae”, while Hintz, who described most of the cerambycids preserved in the MNHNL (Vitali 2010), wrote only about 10 articles. The number of identified species (1100 scarabs vs 900 cerambycids) indicates that both collections were substantially homogeneous (ca 3.5 exemplars per species); thus, they should offer similar probabilities to find new species. The acquisitions of the Museum show that the exotic scarabs were purchased in a slightly higher percentage (28.9% vs 25.2%), but this seems not to be the reason for such a different number of types.

Nonetheless, if the number of specimens collected by Luja in Kondué is compared, the scarabs are much fewer than the cerambycids (831 vs 1660). Luja’s cerambycids represent 51.5% of the exotic specimens while Luja’s scarabs represent only 21.7%. This suggests that Luja collected fewer scarabs, possibly prioritizing aesthetically beautiful but presumably already described species. Considering the relative percentage of scarab species (excluding Passalidae and Lucanidae) conserved in the MNHNL to scarabs worldwide (Ballerio et al. 2010), some outstanding subfamilies look overrepresented: Dynastinae 17.5% (5% worldwide), Cetoniinae 24% (11% worldwide). In contrast, other subfamilies with small or more uniform aspect are underrepresented: Aphodiinae 1.4% (10% worldwide), Melolonthinae 18% (35% worldwide). This choice certainly limited the opportunity to find new species among the scarabs present in the MNHNL.

## Supplementary Material

XML Treatment for Entypophana biapicata

XML Treatment for Metabolus thibetanus

XML Treatment for Autoserica annamensis

XML Treatment for Euphoresia alboparsa

XML Treatment for Hybocamenta ferranti

XML Treatment for Microserica flaveola

XML Treatment for Triodonta lujai

XML Treatment for Trochalus ferranti

XML Treatment for Anomala condophora

XML Treatment for Amaurina ferranti

XML Treatment for Amaurina vittipennis

XML Treatment for Cetonia (Eucetonia) kolbei

XML Treatment for Lomaptera dichropus viridipes

XML Treatment for Cosmovalgus ferranti

XML Treatment for Bilga pictipennis

XML Treatment for Autoserica flaviventris

XML Treatment for Autoserica ruficeps

XML Treatment for Euphoresia kassaiensis
